# Diurnal preference and depressive symptomatology: a meta-analysis

**DOI:** 10.1038/s41598-021-91205-3

**Published:** 2021-06-07

**Authors:** Ray Norbury

**Affiliations:** College of Health, Medicine and Life Sciences, Division of Psychology, Brunel University London, Uxbridge, UB8 3PH UK

**Keywords:** Risk factors, Depression

## Abstract

Eveningness, a preference for later sleep and rise times, has been associated with a number of negative outcomes in terms of both physical and mental health. A large body of evidence links eveningness to Major Depressive Disorder (MDD). However, to date, evidence quantifying this association is limited. The current meta-analysis included 43 effect sizes from a total 27,996 participants. Using a random-effects model it was demonstrated that eveningness is associated with a small effect size (Fisher’s *Z* = − 2.4, 95% CI [− 0.27. − 0.21], *p* < 0.001). Substantial heterogeneity between studies was observed, with meta-regression analyses demonstrating a significant effect of mean age on the association between diurnal preference and depression. There was also evidence of potential publication bias as assessed by visual inspection of funnel plots and Egger’s test. The association between diurnal preference and depression is small in magnitude and heterogenous. A better understanding of the mechanistic underpinnings linking diurnal preference to depression and suitably powered prospective studies that allow causal inference are required.

## Introduction

Circadian rhythms are endogenous processes that follow a near 24-h cycle. These rhythms are self-autonomous, and in humans are controlled by a central oscillator (or master clock) located in the suprachiasmatic nuclei (SCN) of the hypothalamus^1^. Individuals maintained under constant conditions isolated from external timing cues can maintain an endogenous period close to 24 h. However, because these cycles oscillate with periods that differ slightly from 24 h there is a loss of synchrony with the earth’s day-night cycle. To account for this, the central oscillator is adaptable and can be entrained to respond to external time-givers, or zeitgebers (e.g., light). The molecular mechanisms underlying the generation of circadian oscillations relies on multiple proteins generated by clock-related genes that interact to inhibit and activate gene expression in an inhibitory loop that oscillates to produce the near-24-h cycle.

Disruption of circadian rhythms are widely reported in depression. Patients may display a regular daily pattern of symptoms with increased symptom severity often reported in the morning^2^. In MDD there are dampened and phase-shifted rhythms of activity, temperature and hormones (with the exception of cortisol which is increased rather than reduced)^3^ which are correlated with depressive symptom severity^4^. Disrupting circadian rhythms through shift-work, long-distance travel (jet-lag) or misalignment between internal (biological) and social (external) time (referred to as social jet-lag) has been observed to increase depressive symptoms^5–7^. Poor entrainment of the SCN to light (as may occur in Major Depressive Disorder with Seasonal Pattern) and the subsequent disruptions of daily rhythms of hormones and neurotransmitters has been reported as an additional factor to develop depression^8^. In addition, treatments that target circadian rhythms (e.g. agomelatine, early morning bright-light therapy) may be effective in reducing depressive symptoms^9^.

Diurnal preference, or morningness/eveningness, reflects an individual’s preferred timings for sleep and activity and is an individual trait that arises through a combination of endogenous factors and external, environmental stimuli^10^. Evening-types (colloquially referred to as “night owls”) prefer to go to bed late, rise late and plan work and other activities (e.g., meetings, gym visits) for later in the day. By contrast, morning-types (“larks”) prefer to retire early, rise early and plan activities for earlier in the day.

Diurnal preference can be reliably estimated using subjective assessment, which has particular utility for large scale studies. A number of instruments have been developed to measure eveningness and among the most widely used are the Morningness–Eveningness Questionnaire (MEQ)^11^, the reduced version of the MEQ^12^ and the Composite Scale of Morningness (CSM)^13^. All three measures have been shown to have moderate to good reliability and construct validity^14^ and the MEQ has also been demonstrated to be a strong predictor of dim melatonin onset—which is considered the most reliable measure of circadian rhythm in humans^15^.

Twin studies indicate heritability estimates between 46 and 57% for diurnal preference^16^ and diurnal preference is considered a relatively stable trait in adulthood^17^. A better understanding of individual differences in sleep–wake behaviour and how these may relate to disease is becoming increasingly important as a large body of work now suggests that eveningness is associated with a number of negative physical^18^ and mental health outcomes^19,20^ particularly depression^21,22^. Current evidence suggests that eveningness is associated with greater depressive symptomatology^7,22,23^. Eveningness is also associated with having a current diagnosis of depression, treatment for depression or antidepressant use^22,24,25^, non-remission^26^ and suicidal thoughts^27^.

Despite the prevalence and debilitating nature of depression and the overwhelming body of evidence indicating an association between depression and diurnal preference^8–17^ synthesis of these data has been largely restricted to systematic reviews (e.g.^19,20,28,29^). To date, only one study has quantified this relationship in the form of a meta-analysis^30^. The aim of the current study was to extend this previous meta-analysis^30^ (which was limited to data published up to February 2016) to include recent literature published up to 31st December 2020. Based on previous work it is hypothesised that eveningness will be associated with greater depressive symptomatology.

## Methods

The protocol for this meta-analysis was prospectively registered with PROSPERO (CRD4202122977). The raw data and fully reproducible code are available on the OSF (https://osf.io/wyjtx/).

### Literature search

PubMed and Web of Science were searched for articles published between 1st January 2000 and 31st December 2020 using the search terms ("chronotype" OR "diurnal preference" OR "circadian preference" OR "morningness" OR "eveningness" OR "social jetlag") AND ("depression" OR "MDD" OR "Unipolar"). The titles and abstracts of articles returned using this search were initially screened before the full text was examined in greater detail.

To be included in the meta-analysis articles were required to meet the following criteria: (1) Diurnal preference quantified using either the Morning-Evening Questionnaire^11^ the Reduced Morningness–Eveningness Questionnaire^12^ or the Composite Scale of Morningness^13^; (2) Depressive symptomatology measured using either the Beck Depression Inventory (BDI)^31^, the Hospital Anxiety and Depression Scale (HADS)^32^, the Hamilton Rating Scale for Depression (HRSD)^33^, the Depression, Anxiety, Stress Scales (DASS)^34^, the Quick Inventory of Depressive Symptomatology–Self-report (QUIDS-SR)^35^, the Centre for Epidemiology Studies Depression (CES-D)^36^, the Self-Rating Depression Scale (SDS)^37^, the Montgomery-Asberg Depression Rating Scale–Self (MADRS-S)^38^, the Patient Health Questionnaire 9 (PHQ-9)^39^, the Patient Health Questionnaire 4 (PHQ-4)^40^, the Brief Symptom Rating Scale (BSRS)^41^, the Geriatric Depression Scale (GDS)^42^ and the Depressed Mood Scale (DMS)^43^; (3) Sufficient statistical information to estimate an effect size (correlation coefficient, mean and standard deviation or standard error, odds ratio); (4) Written in English; (5) Participants aged 18 or over; (6) For clinical samples, patients diagnosed with MDD; and (5) Published in a peer-reviewed journal. Exclusion criteria were: (1) Studies that included depressive disorders other than MDD (e.g., Seasonal Affective Disorder); and (2) Incomplete or modified versions of the MEQ/rMEQ/CSM (e.g. diurnal preference determined using a single question). Data selection is summarised in Fig. S1, Supplemental Data.

Study quality was evaluated using the McMaster critical review tool for quantitative studies^44^. Data extracted from the included studies was: (1) Authorship; (2) Year of publication; (3) Sample size; (4) Mean age; (5) Age range; (6) Measure of diurnal preference (e.g., MEQ); (7) Depression measure (e.g. BDI); (8) Gender breakdown (e.g. percentage of female participants); and (9) Clinical or non-clinical samples.

### Statistical analyses

All statistical analyses were performed using R version 3.6.1^45^ including the following packages: esc; effectsize; meta; metafor; dmetar: DiagrammeR; DiagrammeRsvg; and ggplot2. Individual effect sizes were obtained from each study. As most studies (52%) reported correlation coefficients, Fisher’s Z transformed correlation coefficient was used as the summary effect size. Odds ratios and standardised mean differences were transformed to Fisher’s Z scores for inclusion in the analysis. The corresponding pooled effect size and its 95% confidence intervals (CI) were calculated from a random-effects model with a Sidik–Jonkman estimator for τ^2^ with Hartung–Knapp adjustment. This method was adopted as a conservative approach in the presence of sample heterogeneity^46^. A pooled effect size of 0.1–0.3 was considered small, 0.3–0.5 medium and 0.5–1 considered a large effect^47,48^. Study heterogeneity was assessed with Q statistics and the *I*^2^ index. Outlier analysis (studies were considered outliers if the 95% CI was outside the pooled effect size 95% CI) were also performed and the random-effects model refitted after excluding any such study. To assess the potential impact of publication bias, funnel plots were visually inspected, and an Egger’s test conducted to quantify asymmetry with a *p* value of < 0.05 considered evidence of asymmetry that may reflect publication bias. Based on the outcome of the Egger’s test, a trim and fill procedure was conducted to impute potential missing studies into the funnel plot to achieve symmetry. Categorial moderators (clinical vs. non-clinical, diurnal preference measure, clinical measure and published in 2020 *vs* any other year (this latter analysis was included as studies published in 2020 overlap with the COVID-19 outbreak and may therefore include data collected during the pandemic) were investigated uses subgroup analyses. Continuous variables (age, year of publication, sample size and percentage of female participants) were explored using meta-regression.

## Results

The initial literature search returned 864 articles (PubMed = 318, Web of Science = 546, see Supplemental S1 for a graphical overview). Following article screening, a total of 51 studies were entered into the initial random effects model. Outlier analyses identified a total of ten studies^49–58^ and these were subsequently excluded from the analysis (See Supplemental S2 for details of these studies including the 95% CI for each excluded effect size). Heterogeneity was reduced after excluding these studies (prior to exclusion *I*^2^ = 77.9%, Q(52) = 235.11, *p* < 0.001, after exclusion *I*^2^ = 60.2%, Q(42) = 105.5, *p* < 0.001) but remained substantial and significant. The final sample included 43 effect sizes from a total 27,996 participants with a mean age of 32 years. The predominant measure of eveningness was the MEQ (59% of included studies), CES-D and BDI where the most common measures of depressive symptomatology (respectively, 26% and 19%) and non-clinical samples made up 86% of effect sizes. Details of the included studies are shown in Table 1.

**Table 1:** Study characteristics. For abbreviations please see main text.

Author	Chronotype	Age	N	Females (%)	Sample	Depression measure
Akram et al.^59^	MEQ	24	453	75	Non-clinical	HADS—depression subscale
Asarnow et al.^60^	CSM	43	139	66	Clinical	HRSD
Aydin et al.^61^	MEQ	22	209	49	Non-clinical	DASS-21 depression subscale
Bakotic et al.^62^	CSM	21	1052	62	Non-clinical	HADS—depression subscale
Berdynaj et al.^63^	MEQ	21	86	78	Non-clinical	DMS
Chan et al.^26^	MEQ	51	253	84	Clinical	BDI
Coleman and Cain^64^	rMEQ	46	424	73	Clinical	HRSD
Furusawa et al.^65^	MEQ	41	362	0	Non-clinical	SDS
Gaspar-Barba et al.^27^	MEQ	32	82	37	Clinical	BDI
Haraszti et al.^66^	MEQ	38	44	100	Non-clinical	PHQ-9
Hidalgo et al.^23^	MEQ	31	142	61	Non-clinical	QUIDS-SR
Hirata et al.^67^	MEQ	22	33	52	Non-clinical	SDS
Horne et al.^53^	rMEQ	24	167	77	Non-clinical	CES-D
Hou et al.^68^	MEQ	37	884	63	Non-clinical	CES-D
Hsu et al.^69^	MEQ	20	790	53	Non-clinical	MADRS
Inomata et al.^70^	MEQ	21	27	48	Non-clinical	CES-D
Jankowski and Dmitrzak-Weglarz^71^	CSM	32	338	51	Non-clinical	HADS—depression subscale
Jankowski^72^	CSM	22	974	70	Non-clinical	PHQ-4—depression subscale
Jeon et al.^73^	MEQ	32	700	91	Non-clinical	CES-D
Kang et al.^74^	rMEQ	19	940	63	Non-clinical	DASS-21 depression subscale
Khan et al.^75^	MEQ	40	59	54	Non-clinical	BSRS
Lau et al.^76^	CSM	21	230	66	Non-clinical	CES-D
Lester^77^	MEQ	22	194	72	Non-clinical	CES-D
Liberman et al.^78^	MEQ	19	242	64	Non-clinical	CES-D
Lin et al.^79^	rMEQ	27	1791	70	Non-clinical	CES-D
Markarian et al.^80^	MEQ	38	296	60	Non-clinical	HADS—depression subscale
Müller et al.^81^	MEQ	42	64	61	Clinical	BDI
Ong et al.^82^	CSM	49	156	NR	Non-clinical	BDI-short form
Park et al.^83^	CSM	19	5632	51	Non-clinical	DASS-21 depression subscale
Park^84^	CSM	37	29	72	Clinical	BDI
Przepiorka et al.^85^	CSM	20	398	71	Non-clinical	BDI
Randler et al.^86^	CSM	22	277	100	Non-clinical	HADS—depression subscale
Selvi et al.^55^*	MEQ	31	80	55	Non-clinical	BDI-II
Smagula et al.^87^	CSM	70	54	70	Non-clinical	PHQ-9
Sun et al.^88^	rMEQ	48	629	NR	Non-clinical	CES-D
Togo et al.^89^	MEQ	41	2669	97	Non-clinical	CES-D
Toomey et al.^90^	MEQ	55	1231	NR	Non-clinical	BDI
Türkoglu and Selvi^91^	MEQ	42	70	83	Non-clinical	CES-D
Üzer and Yücens^58^ *	MEQ	65	70	50	Non-clinical	PHQ-9
Üzer and Yücens^92^	MEQ	22	339	54	Non-clinical	BDI
Watts and Norbury^93^	rMEQ	26	240	79	Non-clinical	BDI
Zhang et al.^94^	MEQ	19	616	65	Non-clinical	PHQ-9
Zhou et al.^95^	MEQ	19	4531	30	Non-clinical	HADS—depression subscale

*Two effect sizes were initially estimated for each of these studies two of which were subsequently identified outliers and excluded from the main random effects model.

**Figure 1:**
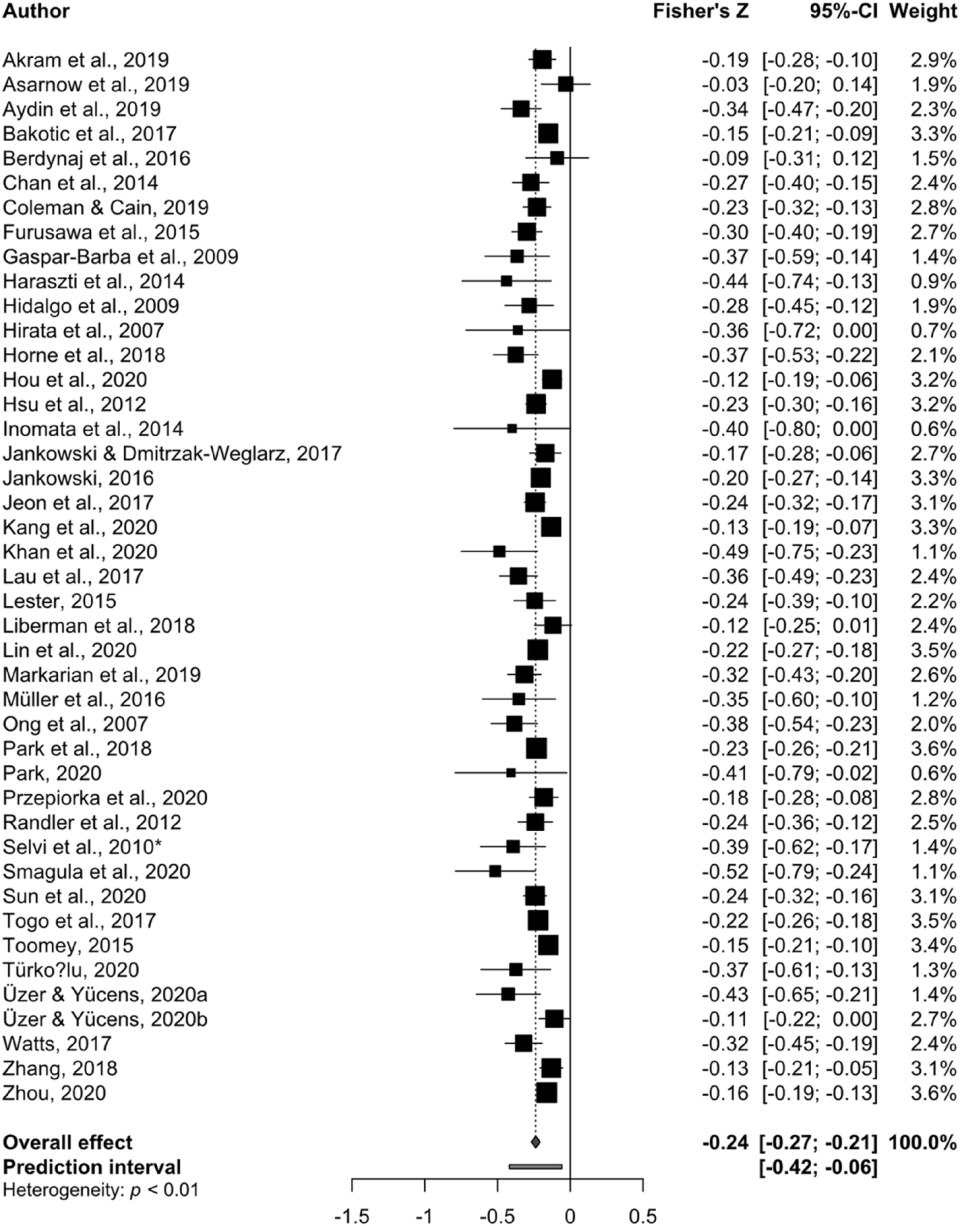
Forest plot of standardised effect sizes from each study. The overall effect is indicated in blue, prediction interval in red.

**Figure 2:**
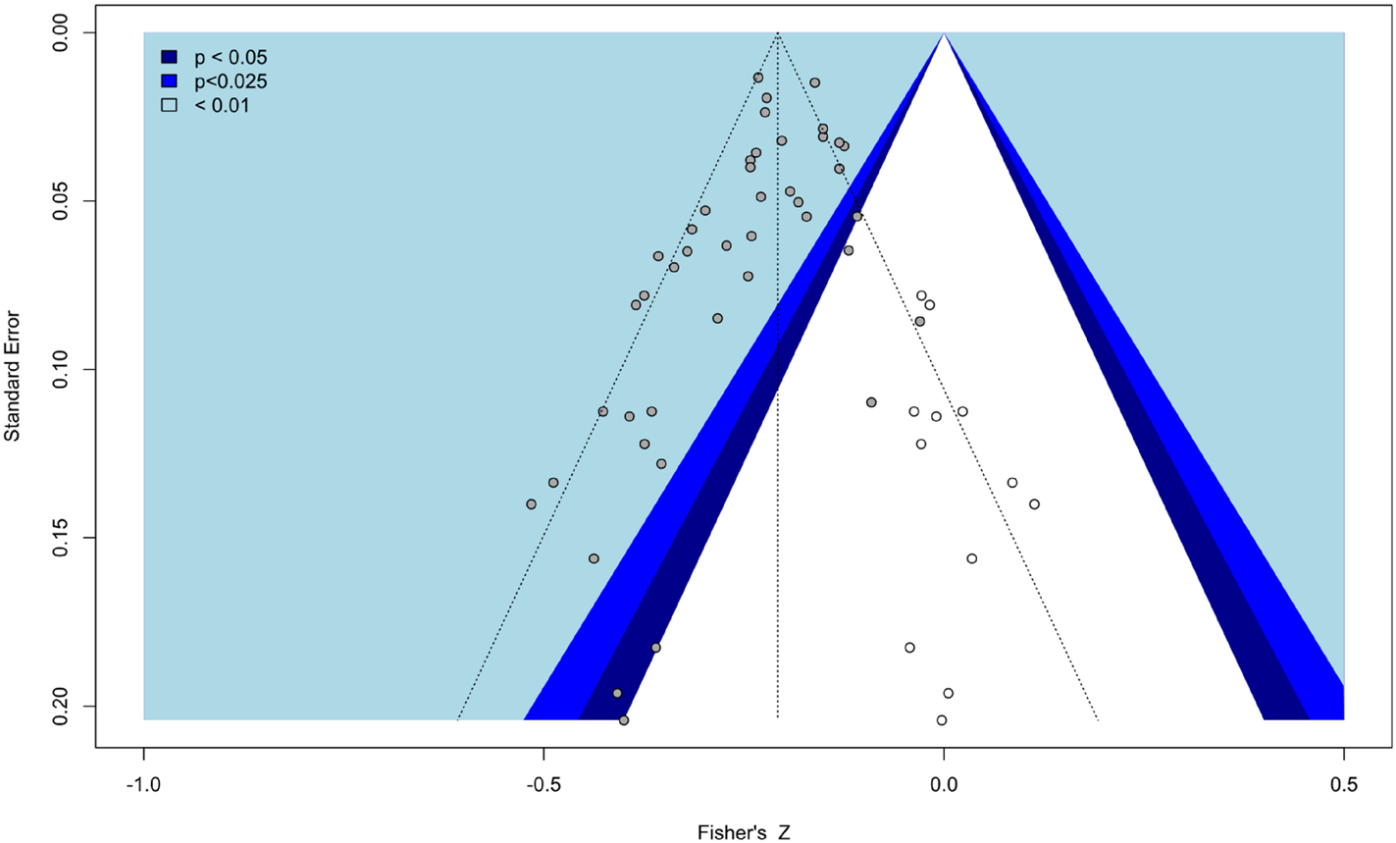
Contour funnel plot indicating potential risk of publication bias. Open dots represent the 12 studies imputed to achieve symmetry.

The effect size estimated from the random-effects model was Fisher’s *Z* = − 0.24, 95% CI [− 0.27. − 0.21], *p* < 0.001, demonstrating a small, but significant, association between eveningness and depression symptomatology (Fig. 1). Visual inspection of the funnel plot (Fig. 2) and the result of the Egger’s test of the intercept (− 1.135, 95% CI [− 1.86, − 0.41], *p* = 0.004) suggest publication bias should be taken into consideration when interpreting the results and the trim and fill adjusted estimate was − 0.21, 95% CI [− 0.2438; − 0.1715], *p* < 0.0001 with twelve adjusted studies.

**Figure 3:**
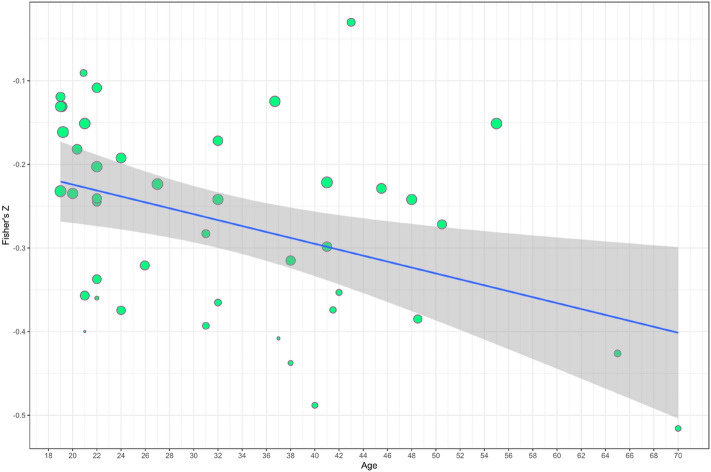
Regression of effect size (Fisher’s *Z*) on age. Line of best fit plus confidence interval, bubbles show study weight.

The substantial heterogeneity between studies suggests a potential impact of moderator variables on the reported association between eveningness and depressive symptomatology. To explore this a series of sub-group meta-analyses were conducted based on four potential confounders: Sample composition (clinical *vs.* non-clinical); Publication year (2020 *vs.* other year); Eveningness measure (MEQ *vs.* CSM *vs.* rMEQ) and Depression measure (BDI *vs.* CES-D *vs.* other). None of the included categorical moderators significantly explained heterogeneity in effect size. A summary of subgroup analyses is presented in Table 2. Meta-regression demonstrated that age was negatively associated with depression symptomatology (β = − 0.003, *p* = 0.03, 95% CI [− 0.005, − 0.0003], R^2^ = 10.63, Fig. 3). Neither sample size (β = 0.00, *p* = 0.12, 95% CI [− 0.00, 0.00], R^2^ = 4.44), year of publication (β = − 0.02, *p* = 0.56, 95% CI [− 0.09, 0.05], R^2^ = 0) or percentage of female participants (β = 0.00, *p* = 0.95, 95% CI [− 0.002, 0.002], R^2^ = 0) were related to the observed association between diurnal preference and depressive symptomatology.

**Table 2:** Subgroup analysis.

Subgroup analyses	Description	Contributing effect sizes	Fishers *Z*	95% CI		*Q*	*p* value
Sample composition	Clinical	7	− 0.27	− 0.37	− 0.17		
	Non-clinical	36	− 0.23	− 0.26	− 0.20	0.52	0.47
Publication year	2020	12	− 0.24	− 0.31	− 0.16		
	Other year^&^	31	− 0.24	− 0.27	− 0.21	0.00	0.94
Eveningness measure	CSM	11	− 0.24	− 0.31	− 0.17		
	MEQ	26	− 0.24	− 0.28	− 0.20		
	rMEQ	6	− 0.24	− 0.30	− 0.17	0.00	0.99
Depression measure	BDI	12	− 0.25	− 0.32	− 0.18		
	CES-D	10	− 0.23	− 0.28	− 0.18		
	Other^&&^	21	− 0.24	− 0.28	− 0.20	0.13	0.94

Other year^&^ = publication year other than 2020; Other^&&^ = any depression measure other than BDI or CES-D (see text for details). Q-test for between study heterogeneity, *p* value for subgroup differences.

## Discussion

The current findings demonstrate a small but significant association between diurnal preference and depressive symptomatology. All of the reported studies indicated a positive association between eveningness and depression, ranging between − 0.52 and − 0.03. The summary effect size for the random effects model was − 0.24 which is largely consistent with an earlier meta-analysis^30^ that reported an effect size of − 0.2 and together these data suggest a small but reliable association between eveningness and depression. Contrary to the findings of Au and Reece, in the current analysis evidence of a potential publication bias (i.e. statistically significant or favourable results being more likely to be published than studies with non-significant or unfavourable results) was observed. The adjusted effect size (Fishers *Z* = − 0.21), however, remained significant. Subgroup analyses demonstrated no moderating effect of sample characteristics, eveningness or depression measure, or studies published in 2020 *vs.* any other year. Meta-regression showed a significant effect of age on the association between eveningness and depression symptomatology, but no evidence for a moderating effect of sample size, gender ratio, or year of publication.

A long-standing question in the literature is one of directionality; does eveningness cause depression or is eveningness a consequence of the disorder? The cross-sectional studies quantified here cannot speak directly to this question. However, the current results demonstrated no significant difference between clinical and non-clinical samples, a finding consistent with Au and Reece^30^. Eveningness may therefore represent a risk-factor for depression rather than a consequence of the depressed state. The vulnerability-stress hypothesis of depression^96,97^ proposes that depression emerges through an interaction between psychological vulnerability factors (e.g., negative biases/preferential processing of negative material) and an environmental stressor (e.g., bereavement, financial insecurity). Importantly, previous work suggests that eveningness is associated with aspects of negative thinking (i.e. psychological vulnerability factors) in never-depressed individuals. For example, eveningness has been associated with greater recall for negative personality trait words, greater recognition of sad facial expressions^63,98^ and maladaptive emotion regulation strategies^93,99^. Similarly, high neuroticism (i.e. individuals who are emotionally reactive and tend to experience more negative emotions and depression) has also been associated with eveningness^100^. Converging evidence, therefore, suggests that in healthy, never-depressed individuals, eveningness is associated with depressogenic personality types, negative biases in emotional processing and impaired emotion regulation which, if combined with adversity, may lead to depression. These findings also suggest modifiable markers that could be therapeutically targeted to prevent the onset of depression in evening type individuals.

Of the moderators tested here only age was significantly associated with effect size. This contrasts with the findings of Au and Reece (2017) who did not observe a similar relationship. The mean age range in the current study was 19–70, which is broader than included by Au and Reece (19–55, MDD sample only) which may account for the discrepancy. Although it should be noted that for the majority of studies included here (~ 50%) the mean age was less than 30 years of age. Of note, Kim et al. recently reported no difference in prevalence rates for depression in late chronotypes *vs.* neither types in a population of Korean adults stratified by age (19–40, 41–59 and 60–80 years). However, although the total sample size was large (*N* = 6382) the number of participants in the older 60–80 years group classified as evening-type was small (*N* = 22) which may limit interoperability^101^. Counter to this, eveningness has been associated with increased odds for reporting depression in a large sample of older adults (age range 40–70 years) taken from the UK Biobank^102^. Similarly, here increasing age was associated with increased depressive symptomatology but the factors underpinning this effect remain to be elucidated. Older individuals that remain more evening-type may gradually lose friendship networks and group allegiances as peers gravitate to a social schedule in synchrony with their changing circadian typology, potentially leaving evening-prone individuals more isolated and potentially more prone to depression. This notion, however, is purely speculative and requires further investigation with suitably powered, prospective studies to determine the potential impact of age on the association between eveningness and depression.

There are several limitations associated with this work which should be considered when interpreting the results. A general limitation of meta-analyses is that by creating a summary of outcomes, important between-study differences are ignored. To formally address this here study inclusion was restricted to adults, for clinical samples mood disorders other than MDD were excluded and only studies that used validated instruments to measure depressive symptomatology and diurnal preference were included. In addition, moderator analysis and meta-regression were employed to explore study heterogeneity. More specifically, the current analysis was unable to account for important factors that may impact the results. Sleep duration and/or sleep quality, for example, were not taking into consideration (zero-order correlations or unadjusted odds-ratios/mean differences were reported). Similarly, social jet-lag, the difference between internal rhythm and external demands (e.g. work or university), which may be more pronounced in evening-types and is associated with increased likelihood of reporting depressive symptoms^103,104^ was not included in this meta-analysis. The current report, therefore, cannot directly assess the potential impact of social jetlag on the association between eveningness and depressive symptoms. Further, the terms chronotype and diurnal preference are frequently used interchangeably in the literature but reflect different aspects of the same phenomenon. Here, the focus was diurnal preference and the questionnaires included limited to the MEQ, rMEQ and CSM which determine morningness/eveningness preferences based on self-reported preferences for times of activity and rest. These measures, therefore, reflect a personality trait. By contrast, instruments such as the Munich Chronotype Questionnaire (MCTQ)^105^ measure behaviour (mid-point of sleep on free days) which can be viewed as an indicator of state^106^. The focus of the current report was unipolar depression, but increasing evidence links eveningness with other affective disorders such as bipolar disorder^107^ and Major Depressive Disorder with Seasonal Pattern^108^ and anxiety^109^. Future meta-analyses that review and synthesise the recent literature related to these disorders is warranted. Finally, it should also be noted that all phases of this review and analyses were conducted solely by the author.

In summary, the current meta-analysis demonstrated that eveningness is associated with depressive symptoms. These data are largely consistent with a previous meta-analysis^30^ and the extant literature. The underlying causes that lead to depression are likely multifactorial and progress in understanding the links between diurnal preference and depression is predicated on a better understanding of the mechanistic underpinnings and suitably powered prospective studies that allow causal inference.

## Supplementary Information

Supplementary Information 1.

Supplementary Information 2.

Supplementary Information 3.

Supplementary Information 4.
