# Supplementary material for: Diurnal preference and depressive symptomatology: a meta-analysis
Source: Sci Rep. 2021 Jun 7;11:12003. doi: 10.1038/s41598-021-91205-3 (PMC8184740; doi:10.1038/s41598-021-91205-3)
Supplement: Supplementary file 1 — Supplementary Information 1. [file 41598_2021_91205_MOESM1_ESM.docx]

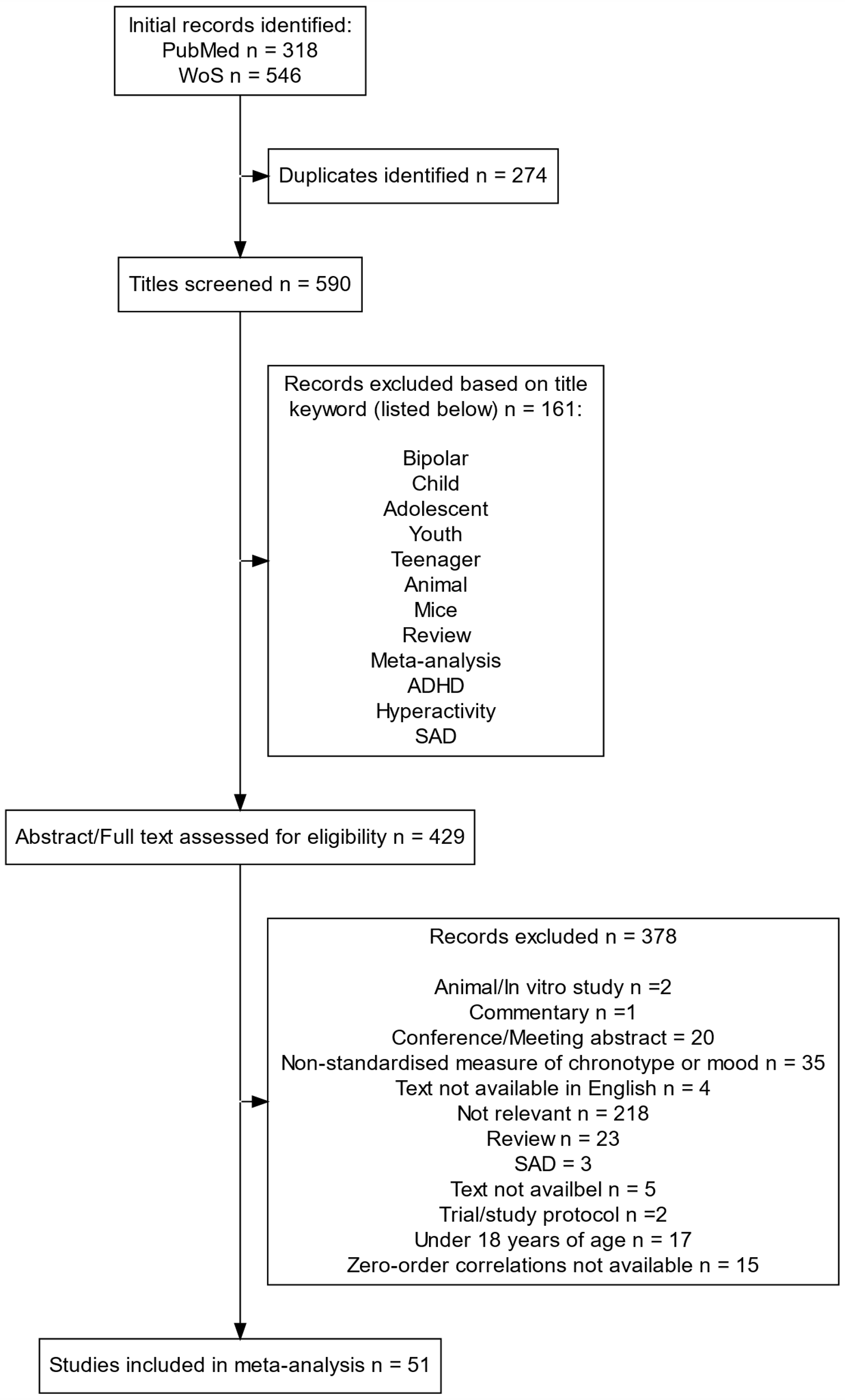


**Figure S1.** Graphical overview of the study selection process (Wos: Web of Science, SAD = Seasonal Affect Disorder).
