# Supplementary material for: Diurnal preference and depressive symptomatology: a meta-analysis
Source: Sci Rep. 2021 Jun 7;11:12003. doi: 10.1038/s41598-021-91205-3 (PMC8184740; doi:10.1038/s41598-021-91205-3)
Supplement: Supplementary file 2 — Supplementary Information 2. [file 41598_2021_91205_MOESM2_ESM.docx]

| Study | Effect size Confidence Interval |
| --- | --- |
| Bacaro et al., 2020 | [-0.1537; -0.0658] |
| Choi et al., 2019 | [-0.1099; 0.0811] |
| Corruble et al., 2014 | [-0.1563; -0.0100] |
| Hogben et al., 2007 | [-0.1563; -0.0100] |
| Horne & Norbury, 2018 | [-0.1142; 0.4576] |
| Hwang et al., 2016 | [-0.1112; 0.1388] |
| Selvi et al., 2010 | [-0.9809; -0.5341] |
| Sultan et al., 2020 | [-0.1422; -0.0181] |
| Thapa et al., 2020 | [-0.8716; -0.4776] |
| Üzer & Yücens, 2020 | [-0.1381; 0.3408] |

**Table S2.** Studies identified as outliers and excluded from subsequent analyses. The pooled effect size and confidence interval for the initial random effects model *N* = 53) was -0.2295 [-0.2729; -0.1862]. For the above studies the individual 95% CI was outside this initial pooled effect size 95% CI.
