# Supplementary material for: Diurnal preference and depressive symptomatology: a meta-analysis
Source: Sci Rep. 2021 Jun 7;11:12003. doi: 10.1038/s41598-021-91205-3 (PMC8184740; doi:10.1038/s41598-021-91205-3)
Supplement: Supplementary file 4 — Supplementary Information 4. [file 41598_2021_91205_MOESM4_ESM.docx]

| Author,date | Was the purpose of the study clearly stated? | Was the relevant background literature reviewed | Sample details | | Outcomes | | Results | | Appropriate conclusions drawn |
| --- | --- | --- | --- | --- | --- | --- | --- | --- | --- |
|  |  |  | Sample details described | Justification for sample size | Reliable | Valid | Statistical significance reported | Analysis appropriate |  |
| Akram et al., 2019 | ✓ | ✓ | ✓ | NR | ✓ | ✓ | ✓ | ✓ | ✓ |
| Asarnow et al., 2019 | ✓ | ✓ | ✓ | NR | ✓ | ✓ | ✓ | ✓ | ✓ |
| Aydin et al., 2019 | ✓ | ✓ | ✓ | NR | ✓ | ✓ | ✓ | ✓ | ✓ |
| Bakotic et al., 2017 | ✓ | ✓ | ✓ | NR | ✓ | ✓ | ✓ | ✓ | ✓ |
| Berdynaj et al., 2016 | ✓ | ✓ | ✓ | NR | ✓ | ✓ | ✓ | ✓ | ✓ |
| Chan et al., 2014 | ✓ | ✓ | ✓ | NR | ✓ | ✓ | ✓ | ✓ | ✓ |
| Coleman & Cain, 2019 | ✓ | ✓ | ✓ | NR | ✓ | ✓ | ✓ | ✓ | ✓ |
| Furusawa et al., 2015 | ✓ | ✓ | ✓ | NR | ✓ | ✓ | ✓ | ✓ | ✓ |
| Gaspar-Barba et al., 2009 | ✓ | ✓ | ✓ | NR | ✓ | ✓ | ✓ | ✓ | ✓ |
| Haraszti et al., 2014 | ✓ | ✓ | ✓ | NR | ✓ | ✓ | ✓ | ✓ | ✓ |
| Hidalgo et al., 2009 | ✓ | ✓ | ✓ | NR | ✓ | ✓ | ✓ | ✓ | ✓ |
| Hirata et al., 2007 | ✓ | ✓ | ✓ | NR | ✓ | ✓ | ✓ | ✓ | ✓ |
| Horne et al., 2018 | ✓ | ✓ | ✓ | NR | ✓ | ✓ | ✓ | ✓ | ✓ |
| Hou et al., 2020 | ✓ | ✓ | ✓ | NR | ✓ | ✓ | ✓ | ✓ | ✓ |
| Hsu et al., 2012 | ✓ | ✓ | ✓ | NR | ✓ | ✓ | ✓ | ✓ | ✓ |
| Inomata et al., 2014 | ✓ | ✓ | ✓ | NR | ✓ | ✓ | ✓ | ✓ | ✓ |
| Jankowski & Dmitrzak-Weglarz, 2017 | ✓ | ✓ | ✓ | NR | ✓ | ✓ | ✓ | ✓ | ✓ |
| Jankowski, 2016 | ✓ | ✓ | ✓ | NR | ✓ | ✓ | ✓ | ✓ | ✓ |
| Jeon et al., 2017 | ✓ | ✓ | ✓ | NR | ✓ | ✓ | ✓ | ✓ | ✓ |
| Kang et al., 2020 | ✓ | ✓ | ✓ | NR | ✓ | ✓ | ✓ | ✓ | ✓ |
| Khan et al., 2020 | ✓ | ✓ | ✓ | NR | ✓ | ✓ | ✓ | ✓ | ✓ |
| Lau et al., 2017 | ✓ | ✓ | ✓ | NR | ✓ | ✓ | ✓ | ✓ | ✓ |
| Lester, 2015 | ✓ | ✓ | ✓ | NR | ✓ | ✓ | ✓ | ✓ | ✓ |
| Liberman et al., 2018 | ✓ | ✓ | ✓ | NR | ✓ | ✓ | ✓ | ✓ | ✓ |
| Lin et al., 2020 | ✓ | ✓ | ✓ | NR | ✓ | ✓ | ✓ | ✓ | ✓ |
| Markarian et al., 2019 | ✓ | ✓ | ✓ | NR | ✓ | ✓ | ✓ | ✓ | ✓ |
| Müller et al., 2016 | ✓ | ✓ | ✓ | NR | ✓ | ✓ | ✓ | ✓ | ✓ |
| Ong et al., 2007 | ✓ | ✓ | ✓ | NR | ✓ | ✓ | ✓ | ✓ | ✓ |
| Park et al., 2018 | ✓ | ✓ | ✓ | NR | ✓ | ✓ | ✓ | ✓ | ✓ |
| Park, 2020 | ✓ | ✓ | ✓ | NR | ✓ | ✓ | ✓ | ✓ | ✓ |
| Przepiorka et al., 2020 | ✓ | ✓ | ✓ | NR | ✓ | ✓ | ✓ | ✓ | ✓ |
| Randler et al., 2012 | ✓ | ✓ | ✓ | NR | ✓ | ✓ | ✓ | ✓ | ✓ |
| Selvi et al., 2010* | ✓ | ✓ | ✓ | NR | ✓ | ✓ | ✓ | ✓ | ✓ |
| Smagula et al., 2020 | ✓ | ✓ | ✓ | NR | ✓ | ✓ | ✓ | ✓ | ✓ |
| Sun et al., 2020 | ✓ | ✓ | ✓ | NR | ✓ | ✓ | ✓ | ✓ | ✓ |
| Togo et al., 2017 | ✓ | ✓ | ✓ | NR | ✓ | ✓ | ✓ | ✓ | ✓ |
| Toomey, 2015 | ✓ | ✓ | ✓ | NR | ✓ | ✓ | ✓ | ✓ | ✓ |
| Türko?lu, 2020 | ✓ | ✓ | ✓ | NR | ✓ | ✓ | ✓ | ✓ | ✓ |
| Üzer & Yücens, 2020a | ✓ | ✓ | ✓ | NR | ✓ | ✓ | ✓ | ✓ | ✓ |
| Üzer & Yücens, 2020b | ✓ | ✓ | ✓ | NR | ✓ | ✓ | ✓ | ✓ | ✓ |
| Watts, 2017 | ✓ | ✓ | ✓ | NR | ✓ | ✓ | ✓ | ✓ | ✓ |
| Zhang, 2018 | ✓ | ✓ | ✓ | NR | ✓ | ✓ | ✓ | ✓ | ✓ |
| Zhou, 2021 | ✓ | ✓ | ✓ | NR | ✓ | ✓ | ✓ | ✓ | ✓ |

Table S4. Adapted critical review form -Quantitative studies. NR = Not reported.
